# Case report: An autopsy report of patient with metastatic brain tumor and carcinomatous meningitis mimicking paraneoplastic neurological syndrome

**DOI:** 10.3389/fneur.2024.1471668

**Published:** 2024-10-11

**Authors:** Ryota Amano, Azusa Sunouchi, Yuka Yokota, Kunio Mochizuki

**Affiliations:** ^1^Department of Neurology, Fujiyoshida Municipal Medical Center, Yamanashi, Japan; ^2^Department of Pathology, University of Yamanashi, Yamanashi, Japan

**Keywords:** T2 FLAIR hyperintensity on bilateral temporal polar white matter, paraneoplastic neurological syndrome, lung large cell carcinoma, metastatic brain tumor, carcinomatous meningitis

## Abstract

Differential diagnosis of metastatic brain tumor, carcinomatous meningitis, and paraneoplastic neurological syndrome (PNS) can be challenging in atypical cases. When examining patient with increased T2 fluid-attenuated inversion recovery (FLAIR) hyperintensities in the temporal polar white matter, autoimmune encephalitis, including PNS, should be considered. Herein, we report the case of an 85-year-old man with carcinomatous meningitis due to lung large cell carcinoma. He showed disturbance of consciousness, abnormal behavior, incomprehensible speech, and apathy, which suggested brain dysfunction. Magnetic resonance imaging revealed high intensities on the whole cerebellum on a diffusion-weighted image and bilateral T2 FLAIR hyperintensities in the temporal polar white matter. Cerebrospinal fluid analysis and cytology showed elevated total protein levels, pleocytosis, and atypical cells with nuclear enlargement, hyperchromasia, and irregular shape. Autopsy revealed lung large cell carcinoma and its brain metastasis. Tumor cells were disseminated to the central nervous system along the subarachnoid space. Furthermore, plenty of carcinoma cells and peritumoral enlarged perivascular space were observed in the temporal poles. To our knowledge, this is the first report of bilateral T2 FLAIR hyperintensities in the temporal polar white matter caused by carcinomatous meningitis with pathological confirmation. In patient with carcinomatous meningitis, abnormal T2 FLAIR hyperintensities may not be derived from ischemia or tumor invasion to parenchyma.

## 1 Introduction

Metastatic brain tumors and carcinomatous meningitis are two forms of secondary brain involvement that occur when cancer cells spread from a primary tumor located elsewhere in the body to the brain and its surrounding structures. Patients with metastatic brain tumors or carcinomatous meningitis generally have a poor prognosis despite the treatment (1). When examining patients with malignant tumors who present with neurologic symptoms, neurologists should consider paraneoplastic neurological syndrome (PNS) as a differential diagnosis. PNS is an immune-mediated neurological disorder caused by antibodies against intracellular, neuronal surface, or synaptic proteins expressed by cancer cells. Patients with PNS exhibit various neurological symptoms and frequent abnormal intensities on magnetic resonance imaging (MRI) in regions of central nervous systems (2, 3). Although detecting anti-neuronal antibodies can aid in diagnosing PNS, their sensitivities and specificities are not necessarily high. Therefore, the possibility of PNS cannot be ruled out, even if anti-neuronal antibodies are not detected (4).

Generally, carcinomatous meningitis presents with abnormal enhancement of the meninges or cranial nerve on T1-weighted gadolinium imaging, as well as hyperintensities on T2 fluid-attenuated inversion recovery (FLAIR), and hydrocephalus can be observed in carcinomatous meningitis (5). Detecting T2 FLAIR hyperintensities in cerebral white matter by carcinomatous meningitis was exceptional, and, in that case, metastatic brain tumors and its surrounding edema should be considered. The representative disease that shows T2 FLAIR hyperintensities in cerebral white matter in cancer patients is PNS, except for metastatic brain tumors. Differential diagnosis of metastatic tumor, carcinomatous meningitis, and PNS is not difficult in typical cases; however, it is challenging in atypical cases.

Herein, we reported a case of metastatic brain tumor and carcinomatous meningitis in a patient with lung large cell carcinoma. MRI revealed high intensities on the whole cerebellum on diffusion-weighted imaging (DWI) and hyperintensities in the bilateral temporal polar white matters on T2 FLAIR. At autopsy, lung large cell carcinoma and its metastasis were found. Tumor cells had disseminated throughout the central nervous system along the subarachnoid space. However, no inflammatory cell infiltration into the brain parenchyma was observed in the temporal poles, suggesting a tumor immunity to cancer cells rather than autoimmune encephalitis, including PNS. This is the first report demonstrating bilateral T2 FLAIR hyperintensities in the temporal polar white matters caused by carcinomatous meningitis with pathological confirmation.

## 2 Case description

An 85-year-old man presented to our hospital with an altered consciousness and an abnormal shadow on the right hilar region. He exhibited abnormal behavior, incomprehensible speech, and apathy, for at least 7 days before admission, which suggested brain dysfunction. Upon admission, neurological examination confirmed altered consciousness [Glasgow Coma Scale of 9 (E4V1M4)], with nuchal rigidity and slightly decreased deep tendon reflexes in the lower limbs.

Initial laboratory examinations revealed slightly high serum levels of C-reactive protein (CRP: 0.91 mg/dL) and indicated dehydration and renal failure (blood urea nitrogen: 56.6 mg/dL, creatinine: 1.54 mg/dL). Tumor-specific laboratory tests revealed high serum levels of neuron-specific enolase (NSE: 59.6 ng/mL).

Cerebrospinal fluid (CSF) analysis revealed pleocytosis with mononuclear cell predominance (40 leucocytes/μL with 80% of mononuclear leucocytes) and increased total protein levels (TP; 244 mg/dL) with decreased glucose levels (glucose; 24 mg/dL). CSF cytology showed atypical cells with nuclear enlargement, hyperchromasia, and irregular shape, which suggested carcinomatous meningitis.

**Figure 1:**
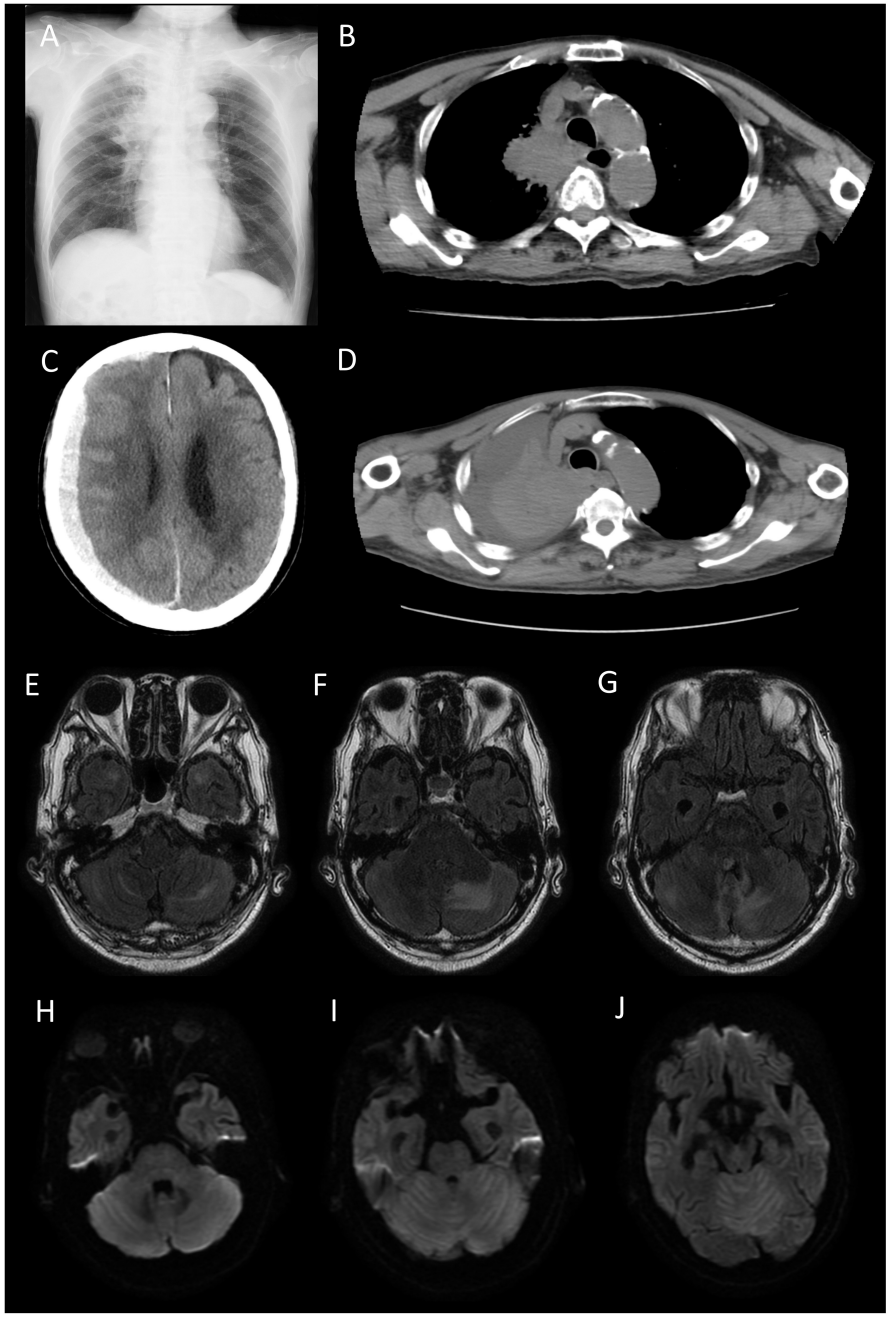
Chest radiograph **(A)** and computed tomography (CT) scan **(B)** on admission. A right hilar tumorous lesion was observed. Brain CT **(C)** and chest CT **(D)** on 10 days after admission. Cryptogenic acute subdural hematoma and increased pleural effusion were apparent. Fluid-attenuated inversion recovery (FLAIR) **(E–G)** and Diffusion-weighted image (DWI) **(H–J)** on admission. FLAIR showed T2 hyperintensities in the bilateral temporal polar white matter and light-predominant edematous cerebellar lesions. DWI showed high intensities in the whole cerebellum but no apparent abnormalities in the bilateral temporal poles. No tumorous lesions were apparent.

Chest radiography and computed tomography (CT) revealed a right hilar tumorous lesion (Figures 1A, B). Brain MRI revealed T2 FLAIR hyperintensities in the bilateral temporal polar white matter and left-predominant edematous cerebellar lesions (Figures 1E–G). DWI showed high intensities on the whole cerebellum but no apparent abnormalities in bilateral temporal poles (Figures 1H–J). No tumorous lesions were apparent, and contrast-enhanced MRI could not be performed because of renal failure.

According to the MRI findings, we considered the possibility of PNS; however, paraneoplastic screening by a fixed tissue-based assay using rat hippocampus and cerebellum (Euroimmune) showed no significance. Moreover, EUROLINE PNS12 Ag (Euroimmune) only showed a weak positive between 6 and 10, suggesting a low titer of anti-recoverin antibodies, an autoantibody typically associated with autoimmune retinopathy.

He received two courses of high-dose methylprednisolone over 2 weeks (1,000 mg/day × 3 days intravenously as one course per week); however, increased pleural effusion and cryptogenic acute subdural hematoma were observed 10 days after admission (Figures 1C, D). Despite the palliative care, he died 15 days after admission.

## 3 Postmortem pathological findings

**Figure 2:**
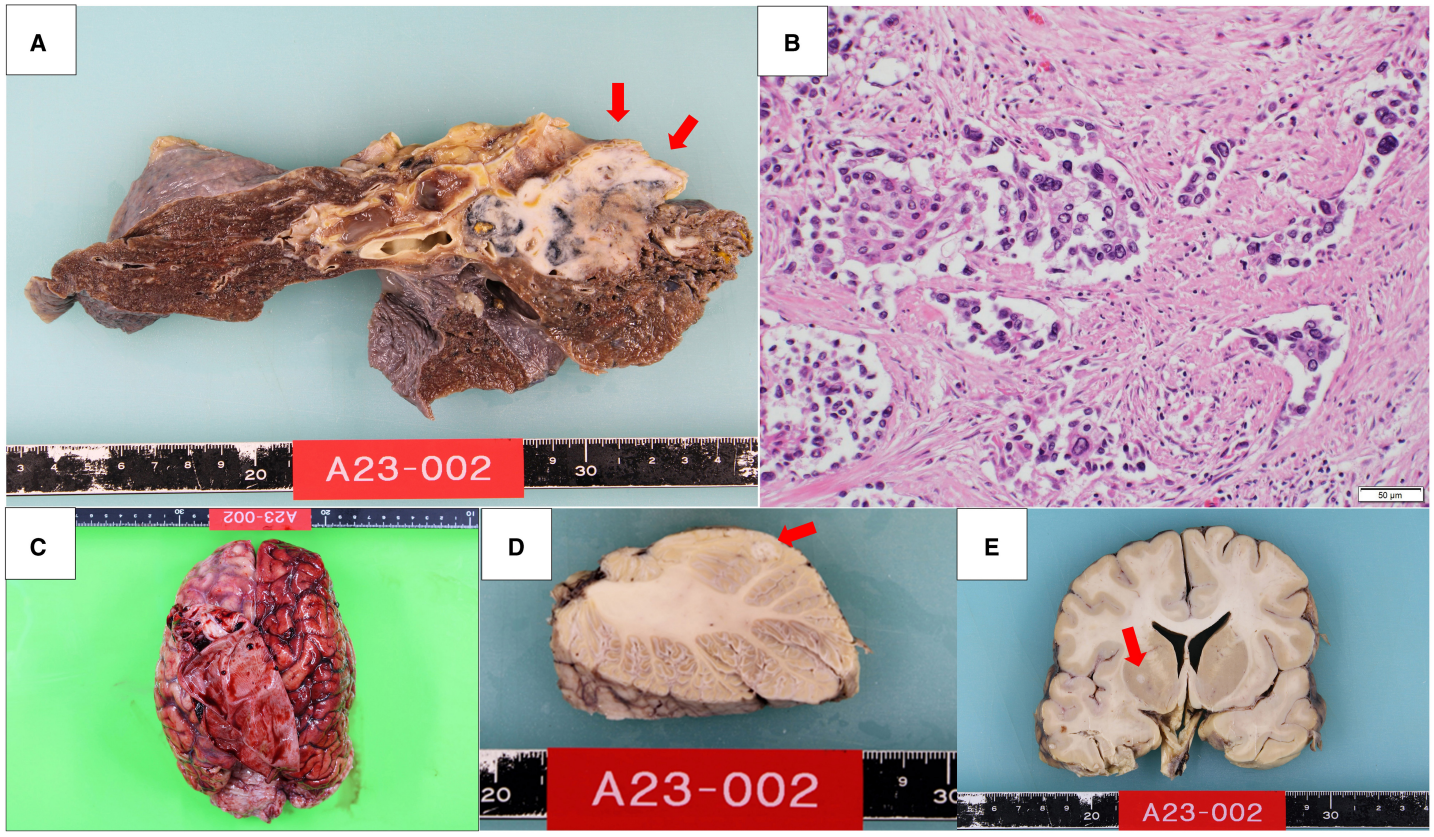
A 6.0 cm diameter tumor was found in the pulmonary hilum of the right upper lobe of the lung **(A)**. Histologically, cells with a high nucleocytoplasmic ratio and large polygonal cells proliferated in a solid or a trabecular pattern **(B)**. Macroscopic features of the brain **(C)**. There was a 6 mm diameter nodule in the left cerebellum **(D)** and a 4 mm diameter nodule in the right globus pallidus **(E)**.

An autopsy was performed 23 h and 40 min after his death. A tumor measuring 6.0 cm diameter was identified in the pulmonary hilum of the right upper lobe of the lung (Figure 2A). Histological examination revealed cells with a high nucleocytoplasmic ratio and large polygonal cells, proliferating in a solid or a trabecular pattern, with a mixture of cells with well-defined nucleoli (Figure 2B). Extensive necrosis was observed within the tumor area. Immunohistochemical analysis of tumor cells revealed the following results: AE1/AE3(+), TTF-1(–), p40(–), chromogranin A(–), synaptophysin(–), CD56(–), INSM1(–). Mitotic counts reached up to 15 mitoses/high-power field (HPF), with an overall Ki67 labeling index of ~5% and up to 40% for hot spots. Based on these findings, we diagnosed the case as large cell carcinoma (null immunophenotype) (6). Additionally, lymph node metastasis and bilateral adrenal metastasis were present.

The brain weighed 1,270 g and showed a subdural hematoma (Figure 2C). There was a 6 mm diameter nodule in the left cerebellum and a 4 mm diameter nodule in the right globus pallidus, which were histologically identified as brain metastases of lung cancer (Figures 2D, E). Additionally, tumor cells were found infiltrating the subarachnoid space, indicating meningeal dissemination. The meningeal dissemination extended over the surface of the entire central nervous system, including the cerebrum, brainstem, cerebellum, pituitary gland, and spinal cord. The cause of the subdural hematoma was considered to be vascular rupture on the brain surface due to tumor cell infiltration.

**Figure 3:**
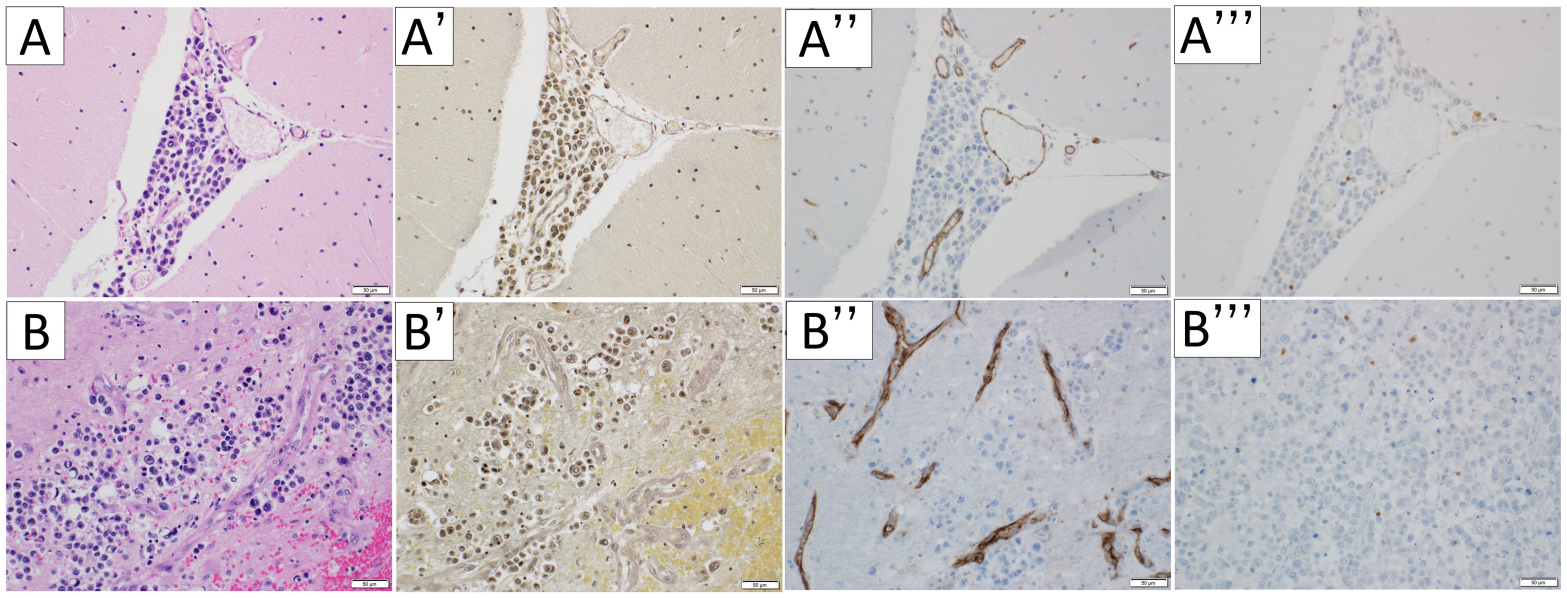
Microscopic appearance of the cerebellum. Hematoxylin-eosin staining **(A, B)**, Elastica van Gieson stain **(A', B')**, immunohistochemical staining for CD31 (**A”, B”)**, and CD3 **(A”', B”')** were performed respectively. In the cerebellum, the tumor extended into the perivascular space **(A)**. The vessels the tumor invades are arteries **(A', A”)**. CD3-immunopositive lymphocytes were limited to the tumoral area **(A”', B”')**. The tumor extended the brain parenchyma through the perivascular space **(B)** and invalid arteries **(B', B”)**. The scale bar length is 50 μm.

In the cerebellum, the tumor extended into the perivascular space (PVS) (Figure 3A) and invaded arteries (Figures 3A', A”). CD3-immunopositive lymphocytes were limited to the tumoral area (Figure 3A”'). Moreover, the tumor extended the brain parenchyma through the PVS (Figure 3B) and invaded arteries (Figures 3B', B”). CD3-immunopositive lymphocytes were limited to the tumoral area (Figure 3B”').

**Figure 4:**
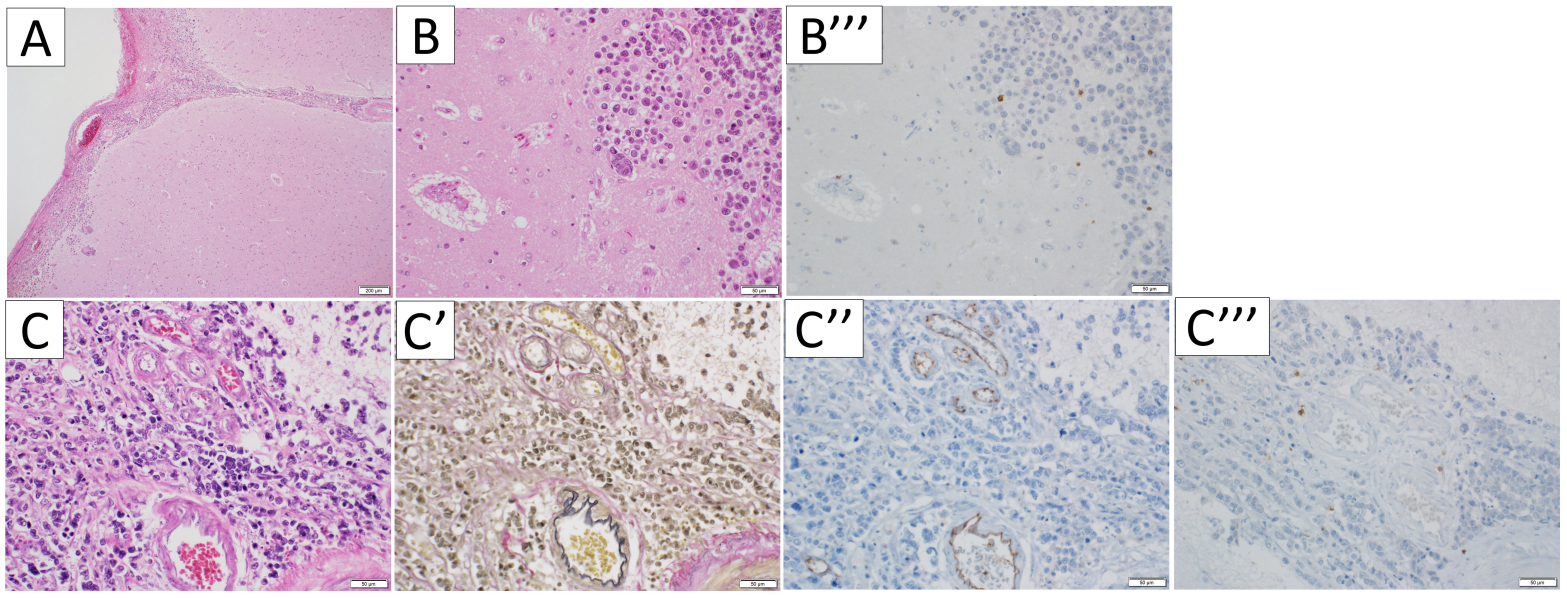
Microscopic appearance of the temporal pole. Hematoxylin-eosin staining **(A, B, C)**, Elastica van Gieson stain **(C')**, and immunohistochemical staining for CD31 **(C”)** and CD3 **(B”', C”')**, were performed respectively. In the temporal pole, the tumor infiltrated along the subarachnoid and perivascular spaces **(A, C)**, and the perivascular spaces were edematous and enlarged **(B)**. The vessels the tumor invades are arteries **(C', C”)**. CD3-immunopositive lymphocytes were limited to the tumoral area **(B”', C”')**.

In the temporal pole, the tumor infiltrated along the subarachnoid space and PVS (Figures 4A, C). The PVS was edematous and enlarged (Figure 4B). The tumor also invaded arteries (Figures 4C', C”). CD3-immunopositive lymphocytes were limited to the tumoral area, with no inflammatory cell infiltration into the brain parenchyma, suggesting tumor immunity to cancer cells rather than autoimmune encephalitis including PNS (Figures 4B”', C”') (7). There was no evidence of neuronal cell loss or gliosis. The infiltrating tumor cells were larger than other sites, and the PVS was edematous and enlarged around the tumor lesion. However, there were no apparent differences between the PVS area of the temporal pole and those of the other regions, except for peritumoral PVS.

## 4 Discussion

In this case, the presence of carcinomatous meningitis was confirmed through CSF cytology, which revealed malignant cells, and paraneoplastic screening using a fixed tissue-based assay in rat hippocampus and cerebellum (Euroimmune) showed no considerable changes. Moreover, EUROLINE PNS12 Ag (Euroimmune) did not detect any antibody that could explain the patient's symptoms. Furthermore, postmortem pathological findings did not support autoimmune mechanisms. Despite this, we considered the possibility of co-existing PNS before patient's death, because T2 FLAIR hyperintensities spread to anatomically distant regions (bilateral temporal polar white matter and cerebellum). This pattern is atypical for carcinomatous meningitis. Contrast enhanced T1 weighted image is useful for differentiating brain metastasis, carcinomatous meningitis, and PNS; however, some patients with carcinomatous meningitis have been reported to show brain stem or cerebral cortical or white matter T2 FLAIR hyperintensities without gadolinium enhancement (8, 9). Therefore, it was difficult to rule out overlapping conditions, even if our patient had received a contrast-enhanced T1 weighted image.

Although T2 FLAIR hyperintensities in temporal polar white matter are especially suggested to be diagnostic for cerebral autosomal dominant arteriopathy or myotonic dystrophy type 1 (10), dilated PVS, which mimic cystic neoplasm on brain MRI, are also known to cause T2 FLAIR hyperintensities in temporal polar white matter (11). In conditions such as cerebral autosomal dominant arteriopathy with subcortical infarcts and leukoencephalopathy (CADASIL) and dilated PVS, T2 FLAIR hyperintensities are believed to result from the inhibition of fluid drainage (11, 12). While these can occur through various mechanisms, their occurrence in other conditions, apart from autoimmune encephalitis and PNS, is rarely reported. Moreover, although T2 FLAIR hyperintensities in the cerebral white matter which are caused by carcinomatous meningitis without pathological confirmation are sometimes observed in daily clinical practice, to our knowledge, they have rarely been reported.

The possible factors that contribute to developing edema (T2 FLAIR hyperintensity) in bilateral temporal polar white matter are as follows: physical blood-brain barrier disruption by tumor infiltration, secretion of cytokines that increase the permeability of blood vessels, inhibition of interstitial and CSF drainage system (glymphatic system), and inflammatory response against tumor cells. In this case, the number of malignant cells was higher in the temporal poles than in other sites. Additionally, PVS, which are potential spaces where layers of connective tissue surround the blood vessels and facilitate the passage of interstitial and CSF of the brain acting as a drainage system, known as the glymphatic system (13, 14), were enlarged around the metastatic regions. Thus, the inhibition of the glymphatic system and the inflammatory response against tumor cells may have contributed to the development of edema.

Ayzenberg et al. reported a case of carcinomatous meningitis with diffuse cortical and white matter hyperintensities on DWI and T2 FLAIR imaging (8). They also reported histopathological findings and considered that perivascular and intravascular infiltration by tumor cells resulted in ischemic cortical and white matter stroke; however, in our case, DWI did not show abnormalities in the temporal polar white matter, suggesting that the mechanism behind T2 FLAIR hyperintensities in the temporal polar white matter may not be due to ischemic stroke. It is difficult to explain the mechanisms of edema developing in the bilateral temporal polar white matter since multiple factors may be involved however, we suspect that the disruption of glymphatic system by tumor cell is at least one contributing factor.

In conclusion, we reported a case of metastatic brain tumor and carcinomatous meningitis mimicking PNS in a patient with lung large cell carcinoma. Although brain MRI, especially in the temporal polar white matter, suggested PNS rather than metastatic brain tumor and carcinomatous meningitis, pathological findings revealed the presence of tumor cells and enlarged PVS, with no evidence of autoimmune mechanisms. This is the first report of bilateral T2 FLAIR hyperintensities in the temporal polar white matter caused by carcinomatous meningitis. Physicians should consider carcinomatous meningitis as a differential diagnosis in patients with cancer who show bilateral T2 FLAIR hyperintensities in the temporal polar white matter.

## Data Availability

The raw data supporting the conclusions of this article will be made available by the authors, without undue reservation.
